# Stay cool and keep moving forwards

**DOI:** 10.1038/s41390-024-03546-0

**Published:** 2024-09-06

**Authors:** Alistair J. Gunn, Joanne O. Davidson

**Affiliations:** Department of Physiology, The University of Auckland, Auckland, New Zealand

## How did we get here?

It has been over 60 years since the first cohort studies of cooling for resuscitation were reported,^1^ and over a quarter of a century since the first large animal studies showed that mild hypothermia could improve neural outcomes after hypoxia-ischemia (HI) in large animals.^2,3^ Subsequent studies systematically dissected the window of opportunity for neuroprotection with hypothermia.^4,5^ This was rapidly followed by the first ever small, extremely cautious randomized safety study of therapeutic hypothermia (TH),^6,7^ which showed that it was generally safe, at least in an intensive care environment. This finding of the general safety of hypothermia confirmed that many of the apparent adverse effects of hypothermia in the newborn such as hypoglycemia and acidosis were actually secondary to HI. Importantly, this study also demonstrated that although the Sarnat examination was developed as a retrospective, multimodal assessment of the evolution of HIE over several days, the clinical examination could be used in isolation to identify infants with moderate to severe hypoxic-ischemic encephalopathy in the first 6 h of life who had a high risk of death or disability.^8^ This evidence base supported the development of multiple large randomized controlled trials that confirmed that within strict parameters TH could improve survival without disability after moderate-to-severe-hypoxic ischemic encephalopathy (HIE).^9^ Based on this evidence, TH is now part of standard care for HIE in high income countries,^10^ and remains the only proven treatment for HIE.^11^

Subsequent preclinical studies and a large clinical trial have shown that the current parameters for neuroprotection are essentially optimal. In near-term fetal sheep, hypothermia started 3 h after cerebral ischemia and continued for 48 h was neuroprotective but less so than cooling for 72 h,^12^ whereas extending hypothermia for 120 h was not associated with further overall improvement in histological or electrophysiological outcomes, and in post-hoc analysis may have reduced protection in some regions.^13^ Further, in term piglets, cooling by 3.5 °C from 2 to 25 h after HI, showed essentially identical protection to cooling by 5 °C, while deep cooling by 8.5 °C was deleterious.^14^ These finding are consistent with a large controlled clinical trial, which showed that a longer duration of cooling for 120 h compared to 72 h or deeper cooling, to 32.0 °C vs 33.5 °C for 72 h, or both, did not reduce the risk of death or moderate or severe disability.^15^

## Where we are now?

It is obviously important to find ways to improve on current treatment. Despite the overall benefit associated with TH, ~29% of infants treated with TH still survive with disability. Given that it seems that current protocols for TH can’t be materially improved, the obvious solution is to add other neuroprotective agents.^16^ In the event, disappointingly, recent large trials of combining other potential neuroprotectants with therapeutic hypothermia have had negative results.^17,18^

A recent well powered randomized controlled trial found that add-on treatment with erythropoietin started within 26 h of birth in infants receiving TH for moderate or severe HIE did not reduce the risk of death or neurodevelopment impairment compared to placebo (RR:1.03; 95% CI, 0.86 to 1.24).^18^ Further, in a phase II imaging biomarker-based randomized trial (TOBY-Xe), inhalation of 30% xenon for 24 h started at a median of 10 h of age in infants receiving TH did not improve either the early readout of the ratio of Lactate to N acetyl aspartate on magnetic resonance spectroscopy or death or moderate or severe disability at 2 years of age.^17,19^ It is likely that in part these disappointing results reflect late initiation of treatment, given that all of the preceding animal studies started add-on therapy, much earlier, within the first 6 h. Further, there is evidence for considerable overlap between the mechanisms of action of these specific agents with the effects of hypothermia to suppress inflammation, programmed cell death, and seizures.^20^

These data naturally do not rule out the possibility that there are additional mechanisms that are not suppressed by TH and so could offer complementary protection in combination. The leading current possibility is melatonin dissolved in ethanol, which augmented a relatively short (12 h) period of TH in term piglets when started at 1 or 2 h after acute HI.^21,22^ Importantly, melatonin with ethanol was independently protective when started 1 h after inflammation sensitized HI.^23^ These data are very encouraging, but suggest that it will be important to start therapy as soon as possible in the latent phase. Further, there is promising evidence in rodents that glucagon-like peptide 1 receptor agonist therapy may offer protection in combination with TH,^24,25^ although the window of opportunity for benefit is still unclear.

## Neonatal encephalopathy in low- and middle-income countries

Of considerable concern, the hypothermia for moderate or severe neonatal encephalopathy in low- and middle-income countries (LMICs) (HELIX) trial in term and near-term neonates found that TH was not effective, and was associated with increased risk of death.^26^ It is interesting to note that in a retrospective single-center study of 182 neonates with HIE treated with hypothermia, those who had acute sentinel events had less severe injury on MRI, and better motor and language outcomes at 18–36 months of age.^27^ In HELIX, only 8% of the babies in the hypothermia group and 13% in the normothermia group had a sentinel event, compared with 25% or more in studies from HICs.^28,29^ The presence of clinical seizures before initiation of hypothermia and the predominance of white matter injury compared to basal ganglia and thalamic injury on MRI suggested that these infants were likely exposed to subacute HI starting well before birth.^26^ Supporting this hypothesis, a case-control study of whole blood transcriptomes from babies with HIE from Italy and in the HELIX trial,^30^ found that the most significant genes associated with those with adverse outcome in Italy were related to acute HI, whereas in the LMIC, they were related to intermittent hypoxia with oxidative stress.

Further, it is important to reflect that the major antecedents of HIE may well be different in different LMICs. The HELIX trial was based in southeast Asia (India, Sri Lanka and Bangladesh). By contrast, in sub-Saharan Africa, there is evidence for high rates of infection/inflammation in infants with HIE.^31^ Thus, a unifying hypothesis for the difficulty in further improving outcomes after HIE is that many infants were exposed to injury in labor, well before birth, so that by the time TH can be started, much of the brain is already in the secondary phase.^32^ It is likely that modifying factors such as exposure to infection/inflammation and chronic placental abnormalities contribute directly or indirectly to more rapid evolution of injury.^33–35^ By contrast, confounding by other neurological causes of neonatal encephalopathy is associated with only a small proportion of cases of HIE.^18^

## How should we move forwards?

It is unlikely that there is a single solution to this difficult problem. It is still possible that there could be a better treatment than hypothermia with greater protection and a wider window of opportunity. Basic biology suggests that this hypothetical intervention would need to target the intranuclear, execution phase of cell death to be effective in the phase of secondary deterioration.^36^

**Fig. 1:**
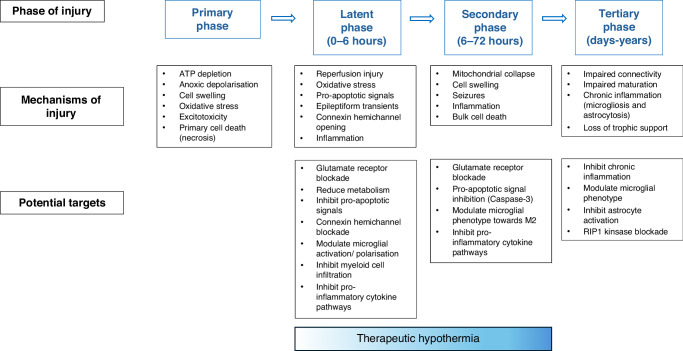
Schematic illustrating the phases of injury after hypoxia-ischemia, and potential therapeutic targets in each phase. The blue bar shows when therapeutic hypothermia needs to be given for neuroprotection, starting in the latent phase, and continued to the end of the secondary phase.

In its absence, broadly speaking we could consider several complementary strategies. First, as well as looking for mechanisms that are not suppressed in the latent and secondary phases by TH, we could explore targeting mediators of cell death and dysmaturation in the tertiary phase after rewarming from TH. This is not improbable. In preterm infants there is already compelling evidence that maternal education and, more broadly, socio-economic factors substantially affect long-term neurodevelopmental outcomes.^37^ Although there is no strong trial evidence for neurobehavioral interventions, these data support their potential. In the term brain, there is growing preclinical evidence for persistent neuroinflammation and cell loss well after TH (see Fig. 1). In near-term fetal sheep, TH for 72 h after cerebral ischemia reduced cell loss and infarction, but only incompletely reduced cortical expression of the pro-inflammatory M1 microglia marker, CD86 on RNAscope.^38^ Clinically, in a cohort of 103 babies with HIE treated with hypothermia, the combined measurements of multiple interleukins within 24 h of birth were highly associated with adverse outcome.^39^ Further, in a small exploratory study, neonatal cytokine levels were associated with subsequent epilepsy.^40^ These data support the hypothesis that persistent upregulation of injurious microglial activity may contribute to partial neuroprotection after hypothermia. Potentially then, targeting this persistent neuroinflammation after rewarming with immunomodulation with strategies such as stem cells,^41,42^ anti-inflammatory agents such as the tumor necrosis factor antagonist, Etanercept,^43,44^ or neurotrophic factors, such as Insulin-Like Growth Factor-I,^45–47^ might further improve the outcomes of therapeutic hypothermia. Clearly, more research is needed!

Finally, the heterogeneity of responses to TH suggests that there may be potential to personalize treatment for babies with HIE. Reliable biomarkers might allow us to better assess whether an individual infant is still in the latent phase when they are likely to respond to hypothermia or have already entered the secondary phase, characterized by delayed onset of seizures and failure of oxidative metabolism, when TH is known to be ineffective.^48^ Alternatively, biomarkers could help assess the presence of inflammation or other markers that might indicate which if any additional treatments may be beneficial. Although to date no biomarkers have been fully established for prospective use in the first 6 h of life, there is a growing body of evidence supporting their potential.^48^ For example, a sub-study of infants with HIE in the Erythropoietin for Hypoxic–Ischemic Encephalopathy in Newborn trial identified a combination of 6 plasma biomarkers measured at different times after birth that was associated adverse outcome.^49^

The single most promising measure so far is the electroencephalogram; it is well understood after HIE in normothermic infants and automated interpretation is becoming more reliable.^50^ It is important to note that TH can alter the prognostic value of the EEG.^51^ In meta-analysis of infants monitored during TH, aEEG had the highest prognostic value at 24 and 72 h and was least predictive 6 h,^52^ suggesting that it is likely to be most useful for prediction of outcome after treatment has been started. Of interest, the CoolCap trial suggested that babies with suppressed EEG background and seizures at the time of randomization did not show significant improvement with hypothermia.^53^ Moreover, in the HELIX trial 74% of TH infants had clinical seizures at the time of randomization.^26^ This suggests that these infants had already entered the secondary phase, or that the injury was too severe to benefit from treatment with TH. Thus, the combination of monitoring for background activity and early onset of seizures in the first 6 h of life may be an effective way of identifying infants who may not benefit from TH, and should be considered for randomization to alternate or complementary interventions, including immunomodulators such as cell therapy.^54^

The obvious corollary is that optimizing the care of high-risk infants is likely to help improve overall outcomes, in partnership between peripheral birth hospitals and those that offer TH. In particular, quality assurance will have an important role by optimizing resuscitation,^55^ avoiding hyperthermia,^28,56^ over-ventilation and hypocarbia,^57,58^ and improving consistency of identification and monitoring of patients with encephalopathy.^59^ Further, hypoglycemia and hyperglycemia during TH have been consistently associated with adverse outcomes.^60,61^ Similarly, as discussed above, EEG/aEEG monitoring has highlighted the association of seizures with outcome.^62^ Moreover, although it is often seen as too hard to study, as highlighted above, there is considerable indirect evidence that the parent-child dyad is critical for long-term outcomes,^37^ and that improving their care, for example by enabling parents to hold their infants during cooling, neurodevelopmental evaluation and interventions, and support for families, particularly deprived families, would have substantial benefits.

In conclusion, continuing focused preclinical and clinical studies will help us to better understand who, and how and, above all, when to treat infants with HIE.
